# The Multi-Purpose Airborne Sensor Carrier MASC-3 for Wind and Turbulence Measurements in the Atmospheric Boundary Layer

**DOI:** 10.3390/s19102292

**Published:** 2019-05-17

**Authors:** Alexander Rautenberg, Martin Schön, Kjell zum Berge, Moritz Mauz, Patrick Manz, Andreas Platis, Bram van Kesteren, Irene Suomi, Stephan T. Kral, Jens Bange

**Affiliations:** 1 Center for Applied Geoscience, Eberhard-Karls-Universität Tübingen, Hölderlinstr. 12, 72074 Tübingen, Germany; martin.schoen@uni-tuebingen.de (M.S.); kjell.zum-berge@uni-tuebingen.de (K.z.B.); moritz.mauz@uni-tuebingen.de (M.M.); manzp@gmx.de (P.M.); andreas.platis@uni-tuebingen.de (A.P.); bram.vankesteren@uni-tuebingen.de (B.v.K.); jens.bange@uni-tuebingen.de (J.B.); 2 Finnish Meteorological Institute, P.O. Box 503, 00101 Helsinki, Finland; irene.suomi@fmi.fi; 3 Geophysical Institute and Bjerknes Centre for Climate Research, University of Bergen, Postbox 7803, 5020 Bergen, Norway; stephan.kral@uib.no

**Keywords:** fixed-wing unmanned aircraft, turbulence measurement, 3D wind vector measurement, stable boundary layer, comparison with measurement tower, unmanned aircraft system (UAS), remotely piloted aircraft (RPA)

## Abstract

For atmospheric boundary-layer (ABL) studies, unmanned aircraft systems (UAS) can provide new information in addition to traditional in-situ measurements, or by ground- or satellite-based remote sensing techniques. The ability of fixed-wing UAS to transect the ABL in short time supplement ground-based measurements and the ability to extent the data horizontally and vertically allows manifold investigations. Thus, the measurements can provide many new possibilities for investigating the ABL. This study presents the new mark of the Multi-Purpose Airborne Sensor Carrier (MASC-3) for wind and turbulence measurements and describes the subsystems designed to improve the wind measurement, to gain endurance and to allow operations under an enlarged range of environmental conditions. The airframe, the capabilities of the autopilot Pixhawk 2.1, the sensor system and the data acquisition software, as well as the post-processing software, provide the basis for flight experiments and are described in detail. Two flights in a stable boundary-layer and a close comparison to a measurement tower and a Sodar system depict the accuracy of the wind speed and direction measurements, as well as the turbulence measurements. Mean values, variances, covariance, turbulent kinetic energy and the integral length scale agree well with measurements from a meteorological measurement tower. MASC-3 performs valuable measurements of stable boundary layers with high temporal resolution and supplements the measurements of meteorological towers and sodar systems.

## 1. Introduction

For atmospheric boundary-layer (ABL) studies, unmanned aircraft systems (UAS) can provide new information in addition to traditional in-situ measurements or ground- and satellite-based remote sensing techniques. Recent developments of UAS and high-performance high-resolution in-situ sensors allow the observation of processes at different levels within the ABL, which so far can only be accomplished by tall meteorological towers or to some extent, although with limited spatial and temporal resolution, by ground based remote sensing systems. The ability of fixed-wing UAS to sample data of the ABL along the flight path supplements ground based measurements and the ability to extend the data horizontally and vertically allows manifold investigations. Representative samples of the ABL can be gathered with a high temporal resolution, or area representative evaluations without the need for multiple measurement platforms. Turbulence along a straight horizontal flight path is not precisely a spatial snapshot, nor a temporally averaged snapshot, but a mixture of both, which can be labeled as quasi-spatial snapshot. The use of such data presumes the following assumptions, pros and cons. The most important compendium is Taylor’s hypothesis of frozen turbulence [1,2] which must be questioned for low frequencies (or low wavenumber) of the spectrum of atmospheric turbulence [3]. Even the inertial subrange of the spectrum according to Reference [4] may not follow Taylor’s hypothesis of frozen turbulence and if eddies of different sizes travel at different velocities, the turbulent wave number spectrum cannot be simply interpreted as the frequency spectrum [5,6]. With aircraft measurements, Taylor’s hypothesis is rather valid, since a long distance is covered within a short time period [7,8]. The downside of transecting the turbulence regime is the shift of the spectrum towards higher frequencies and the need of sensors to be accordingly faster than those of stationary measurement systems. A moving platform in general may be technically more challenging than a stationary measurement system, since the wind vector must be transformed from a moving into an earth bound coordinate system. On the other hand, turbulence measurements along a straight horizontal flight path sampled with a fixed-wing UAS, compared to turbulence measured at a stationary point, enables a faster measurement of the quantity, since the same amount of data can be sampled in shorter time. The UAS moves with its airspeed through the ABL and the measurement at a fixed point samples the air which is advected with the mean flow. This correlation can be beneficial for example, for measurements of transition phases of the ABL, where the state of the ABL changes quickly. The need of statistical significance when calculating turbulence statistics [9] implements further challenges for UAS, because the flight distances along flight paths may be limited due to technical restrictions or legal issues. Generally, heterogeneity of the surface and inhomogeneous footprints of moving and stationary systems also implement difficulties and cause discrepancies for a direct comparison of the two systems. This study aims to validate the measurements of the new mark of the Multi-Purpose Airborne Sensor Carrier (MASC-3) by closely comparing them with measurements from a meteorological tower and subsequently being able to fuse both systems for investigations of stable boundary layers (SBL).

Like micro-meteorological stations, remotely piloted aircraft (RPA) can be equipped with fast and accurate sensors in order to measure atmospheric turbulence. The airframe of the vehicle is referred to as RPA and if the sensor systems and ground control systems are also referred to, the terminology is UAS. UAS can be equipped with similar measurement systems than manned aircraft but are limited by the size of the UAS. Since the beginning of the millennium, the rapid progress in micro-electronics and component miniaturization allowed for a fast development of airframes, autopilots and meteorological sensors for research in the ABL. One of the first low-cost attempts was the remotely-controlled, but not auto-piloted system, KALI, which performed more than 150 flights in Nepal and Bolivia to investigate thermally driven flows modified by orography [10,11]. The following years showed rapidly increasing activities by various research groups, making their sensors and instrumentation airborne within a reasonable budget. Most of those earlier systems are based on fixed-wing airframes as for example, M2AV [12], SUMO [13,14], Smartsonde [15,16], Manta [17], MASC [18], ALADINA [19,20], Pilatus [21] and BLUECAT5 [22]. UAS were used for research in the field of atmospheric physics and chemistry [23,24,25,26], boundary layer meteorology [17,27,28,29,30,31,32,33,34,35,36,37,38], and more recently also to wind-energy meteorology [39,40,41]. The capabilities of UAS for meteorological sampling are broad. The UAS designs range from a more accurate and diverse—but larger—sensor payload, down to small aircraft that can be operated with minimal logistical overhead. Since 2010, the use of rotary-wing multi-copter systems for atmospheric research has increased [38,42,43,44]. With their ability to hover and to slowly ascend and descend vertically, they are the preferred choice for many measurement tasks related to boundary- and surface-layer profiling, but are limited when measuring turbulence.

Measuring the wind speed and direction is a fundamental and elaborate requirement for understanding the processes of the ABL. The common method to measure the turbulent 3D wind vector from research aircraft is a multi-hole probe in combination with an inertial navigation system (INS). By calibration, the pressure readings are used to estimate the airspeed vector of the UAS and with the INS data, multiple coordinate transformations yield the 3D wind vector [45]. This technique originates from manned research aircraft [46] and was adopted by UAS [22,47]. Simplified algorithms to measure the temporally averaged horizontal wind speed and direction such as the “no-flow-sensor” or the “pitot-tube” algorithm, also exist and were compared to the direct 3D wind vector measurement using a five-hole probe by Reference [48].

UAS have the potential to provide new information about the SBL, when applied together with traditional in-situ measurement techniques. Parametrizations of the processes in numerical weather prediction and climate models, yet only apply for stationary and homogeneous surface conditions. The parametrization schemes, for example, the Monin–Obukhov similarity theory (MOST) are known for their shortcomings in characterizing the SBL [49]. Continuous turbulence as a quasi-stationary state may break down and become intermittent. Non-local features such as the stability at higher levels and gravity waves become important, the Coriolis effect and inertial oscillations influence the structure of the SBL and Low Level Jets (LLJ) can develop and generate turbulence by the vertical wind shear [50,51,52,53,54]. For weakly stable boundary layers, transition phases and very stable boundary layers [55] UAS can supplement the limited spatial or temporal coverage of ground-based measurements. On the other hand, SBL conditions also impose challenges for both stationary measurement systems and UAS, since weak turbulent fluxes are difficult to measure and require a high accuracy of the measurement system. Precise and fast measurements of the turbulent 3D wind vector from UAS in combination with meteorological towers and ground-based remote sensing techniques yield new possibilities [56].

The main aim of this study is to validate the turbulent 3D wind vector measurement with MASC-3. To do so, mean values, statistical moments of second order, integral length scales and a spectral analysis can be performed. A comparison to established measurement systems and theory leads towards validation. Firstly, a close comparison with the measurements of a meteorological tower are presented and secondly the data of the tower and the phased array 3D wind Sodar are plotted together with profiles of MASC-3 in a SBL. MASC-3 aims to improve the wind measurement, to gain endurance, to allow operations under an enlarged range of environmental conditions and to enable easy implementation of further sensors by the following measures. The influence on the 3D wind vector measuremnt by the flow field around the aircraft [57] is an important criterion and therefore the new airframe of MASC-3 features a pusher engine in the very back (behind the tail unit) of the UAS as well as a forward-spaced and streamlined sensor hat, where a five-hole probe is mounted (see Section 2.1). Also the flight guidance and the autopilot are of major importance for the 3D wind vector measurement, since the attitude of the UAS, as well as the vehicle velocity, are directly inherited in the calculations. A steady and precise flight of MASC-3 is implemented by the Pixhawk 2.1 “Cube” autopilot (see Section 2.2). The fuselage and the installed sensor hat allow for different payloads, making MASC-3 versatile for many scenarios. The standard payload is described in Section 2.3 and includes an inertial navigation system (INS) Ellipse2-N from sbg-systems [58], a five-hole probe manufactured by the Institute of Fluid Mechanics at the Technische Universität Braunschweig, Germany [59], a fine wire platinum resistance thermometer (FWPRT) developed by Reference [60] and further temperature, surface temperature and humidity sensors. The software architecture is described in Section 2.4 and runs on a Raspberry Pi 3, which allows an easy implementation of future sensors. The in-house developed post-processing software MADA (see Section 2.5) provides a standardized quality control of the gathered data within min after the flight experiment and enables comprehensive quick-looks of mean values and turbulence statistics of the flight experiment.

The measurements of this study were collected during an intensive measurement campaign—”Hailuoto-II”—of the project called Innovative Strategies for Observations in the Arctic Atmospheric Boundary Layer (ISOBAR). The campaign took place over sea ice at the western shore of Hailuoto island in the northern Bothnian Bay on the coast of Finland in February 2018. The main motivation for the ISOBAR project is to develop and apply a new and innovative observation strategy for the stably stratified boundary layer that is based on meteorological UAS, ground-based in-situ and remote-sensing profiling systems [38]. Two flight experiments were dedicated to closely comparing the MASC-3 measurements with the meteorological tower measurements and were conducted in the evening of the 10 February 2018 over the completely frozen bay area of Hailuoto. The methods for the comparison are described in Section 3.2 and are based on a comparative duration of the time series for the stationary and the moving measurement systems, which correspond to the individual fetch of both systems. Section 4.1 compares the measurements by means of time series analysis for mean values of wind speed and direction, variances, turbulent kinetic energy, covariances and integral length scales of the 3D wind vector measurement. The analysis of a fast evolving SBL during the second flight is given in Section 4.2, where the height profiles performed with MASC-3 are supplemented with the tower and Sodar measurements on the ground, illuminating the vast potential of turbulence measurements with MASC-3 in SBL.

## 2. Multi-Purpose Airborne Sensor Carrier—MASC-3

**Figure 1:**
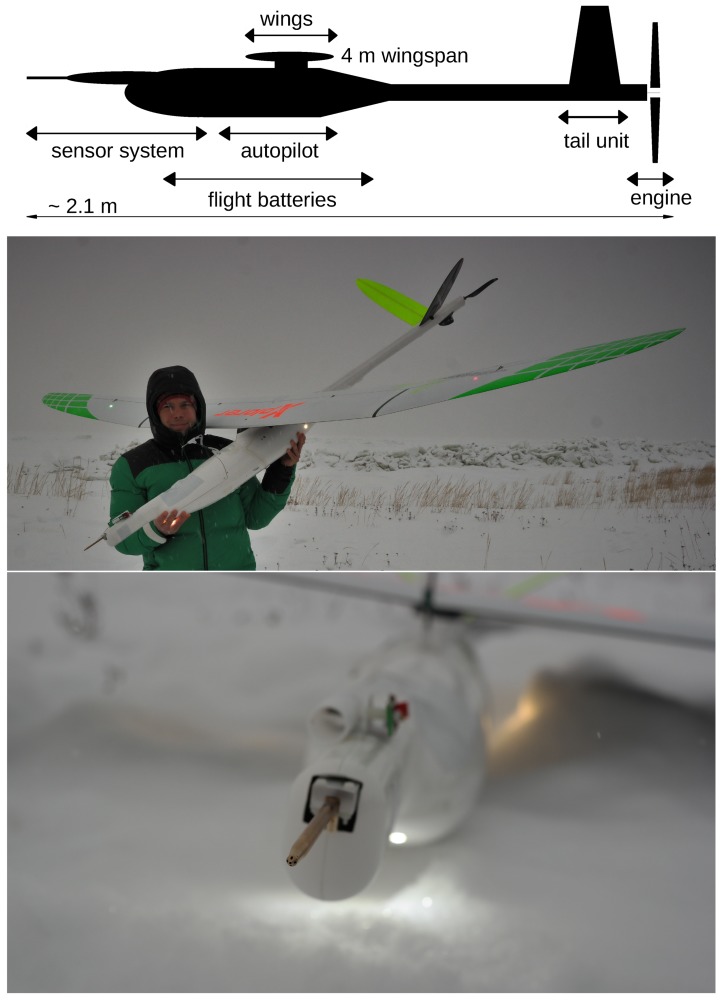
Multi-Purpose Airborne Sensor Carrier (MASC-3) sketch (**top**) and pictures of the airframe with the sensor system (**middle**) and five-hole probe (**bottom**).

A detailed description of the Multi-Purpose Airborne Sensor Carrier (MASC-3) is presented. The design criteria and capabilities of the airframe are given in Section 2.1, followed by a description of the autopilot system Pixhawk 2.1 “Cube” in Section 2.2. The airframe and the autopilot system, as well as the embedded sensor system of MASC-3, were completely reworked compared to the previous version of MASC. The sketch in Figure 1 provides an overview of the new setup. The core of the data acquisition unit is a Raspberry Pi 3, allowing the use of various interfaces to sensor applications, telemetry modules and on-board data processing algorithms. We describe the sensor system in Section 2.3, the data acquisition procedure in Section 2.4 and the post-processing procedure in Section 2.5.

### 2.1. Airframe Design

MASC-3 is a further development of the environment-physics group at the Center for Applied Geo-Science (ZAG), University of Tübingen, Germany and is based on the previous UAS, which was described, for example, in Reference [18,48]. The overall goals for the new design were increasing the accuracy of the wind measurement, gaining endurance, having more flexibility in implementing further sensors in future applications and allowing operations under an enlarged range of environmental conditions. Figure 1 shows the airframe with its sensor nose in the very front of the fuselage. The positioning was chosen in order to be as far away as possible from potential influences on the measurement. Figure 1 shows the sensor system with the five-hole probe, temperature and humidity sensors. Moreover, the engine is positioned in the back, behind the V-tail of the UAV. Due to the significantly increased distance between the measurement system in the nose and the engine position (see Figure 1), compared to the previous version of MASC, potential influences on the measurements are minimized.

The prop wash, vibrations and the magnetic field of the engine are further away from the sensor system. The power unit consists of a highly efficient electrical pusher setup with a gear unit in order to use a large diameter for the propellers, while keeping the engine speed low. The aerodynamic efficiency is high for cruising speeds around 20 ms${}^{-1}$, since a propeller requires large diameters at rather slow drive rates, resulting, with Li-Ion battery packs, in a highly improved overall efficiency of the drive train, compared to the previous version of MASC. Besides, the point of application of the thrust vector has a much smaller lever arm onto the center of gravity compared to the previous MASC with a pusher engine above and behind the main wings, improving the stability of the flight during acceleration and deceleration of the engine. Due to non-zero vertical wind velocity and changes in the horizontal wind speed on turbulent scales, or other motions, for example, thermals or up- and down-drafts due to orography, the aircraft reacts with acceleration or deceleration relative to the air.

To fulfill the requirements of constant altitude, constant flight direction and constant airspeed, the autopilot system of the UAS controls the angle of attack and the throttle. The reactions of the UAS on changes in the wind field, correlate and are proportional to the momentum and the aerodynamic drag of the UAS. Also, the individual flight mechanical behaviour of the UAS and its ability to be susceptible to interaction with turbulence are important. Therefore, the aerodynamic drag must be low and the flight mechanical performance of the wing design with a high lift/drag ratio are very important issues for the precision of the wind and turbulence measurement with five-hole probes [45]. MASC-3 meets these requirements superior to the previous version, since the wings and tail are from an aircraft (XPLORER 3 by NAN Models) of international championships in F3J and F5J glider competitions. The wingspan is 4 m. The streamlined fuselage design offers space for versatile configurations and with the broad range of possible wing loads, the total weight can range from 3.5 kg with a standard measurement setup and small battery capacity, up to ≈8 kg. The maximum flight duration with 18 ms${}^{-1}$ cruising airspeed was proven to be 2 h and is estimated to be 3 h and more. The wings, tail and fuselage are manufactured with fibreglass and carbon fibre composite materials, providing high durability and a light weight construction. With the thermodynamic management of the electrical components, MASC-3 can operate under polar conditions as well as in hot environments. Take-off is performed with a bungee or a winch, if for example, cold temperatures below ≈−10 °C cause the rubber bungee to fail. Trained pilots can land MASC-3 on a strip of less than 10 × 4 m, since large air brakes allow fast descents and precise steering during the approach. High manoeuvrability and a broad range of cruising airspeeds between 14 ms${}^{-1}$ and more than 30 ms${}^{-1}$ allow sampling with high resolution as well as operations in high wind speeds and extreme turbulence.

A new feature of MASC-3 is that it can be equipped with position and strobe lights. Figure 1 shows the lights following the conventions of manned aircraft, allowing take off and landing during night time. As the lighting of MASC-3 fulfills the requirements of the SERA 923/2012 regulation, (see for more details Reference [61]) the aircraft can obtain special permission of the local civil flight authorities for UAS operations during night time and beyond visual line of sight (BVLOS).

**Figure 2:**
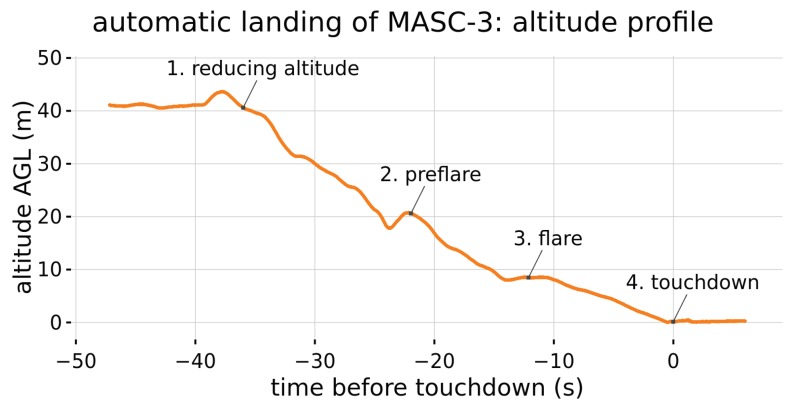
MASC-3 altitude profile during automatic landing procedure.

Since reduced visibility is challenging for the pilot, the flight guidance with the autopilot system PixHawk 2.1 (see also Section 2.2) allows automatic mode just after release from the take-off rope and automatic approach for manual landing procedures or even entirely autonomous landing, as shown in Figure 2.

### 2.2. Flight Guidance, Autopilot System and Flight Patterns

**Figure 3:**
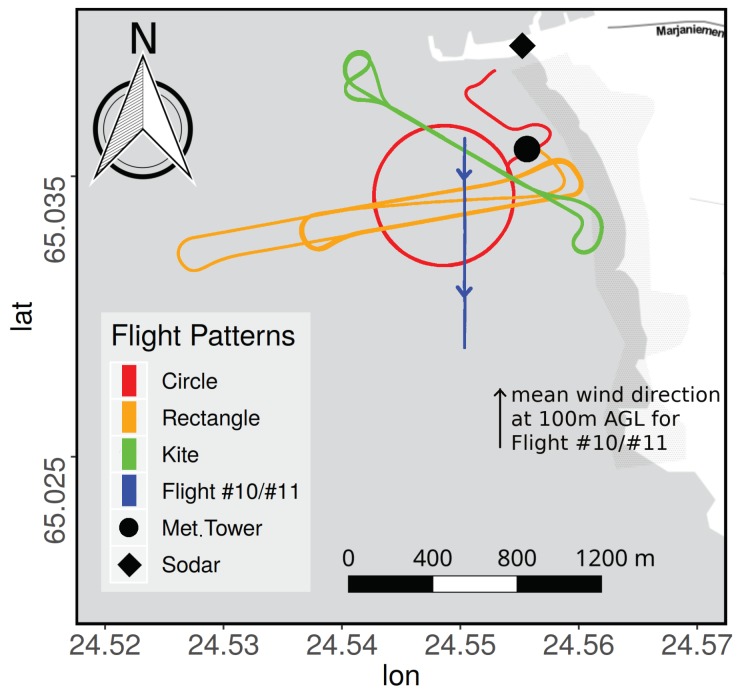
MASC-3 flight paths during the Hailuoto-II campaign. The island is indicated in white color and the grey area indicates water, which was completely frozen during the measurements allowing the installation of the indicated measurement tower. The sodar was installed on the island. The flight paths are plotted from the longitude and latitude readings of the inertial navigation system. The flight path section (leg) of Flight #10 and Flight #11 was used for the comparison between the tower, the Sodar and MASC-3. During Flight #10 and Flight #11 the mean wind direction at 100 m above ground level (AGL) is indicated. Map tiles by Stamen Design (http://stamen.com/) under CC BY 3.0 (http://creativecommons.org/licenses/by/3.0). Data by Open Street Map (http://openstreetmap.org/) under ODbL (http://www.openstreetmap.org/copyright).

When measuring wind and turbulence with a five-hole probe, the UAS needs to be able to repeat a flight pattern over the course of multiple flights to increase the statistical validity of the captured data and to allow for comparisons between different measurement flights. These requirements are met by the autopilot system. Common UAS autopilot systems use an INS (Inertial Navigation System) consisting of one or multiple triple-axis accelerometers, gyroscopes and magnetometers (IMU) for attitude and heading control as well as a GNSS (global navigation satellite system) reciever (GPS, GLONASS, Beidou and/or Galileo) to measure ground speed and location. Some systems may also include a laser altimeter to measure altitude above ground or infrared receivers for communication with ground-based beacons for precision landing. The autopilot system of MASC-3 consists of a Pixhawk 2.1 “Cube” autopilot using a Here+ RTK GPS and magnetometer for position, velocity and heading and a mrobotics MS5525 digital airspeed sensor connected to a pitot-static tube for airspeed measurement. The heated IMU of the Cube allows MASC-3 to operate reliably in very low temperatures and the RTK GPS improves location accuracy over standard GNSS solutions. The Cube is running the open-source Ardupilot autopilot firmware and flight patterns can be programmed before take-off or wirelessly during the flight. Figure 3 shows the flight patterns used for MASC-3 and performed during the ISOBAR campaign Hailuoto-II. The ”Rectangle” pattern is the most common one with MASC-3, performing long up- and downwind measurement legs. A rectangle (also called racetrack) is repeated several times at one altitude and one measurement flight normally consists of several racetracks at different altitudes. The up- and downwind portions of one racetrack are called measurement legs. The track marked as Flight #10/#11 shows the flight path of the upwind legs of a rectangle pattern that is used for the comparison in Section 4. The length of the flight legs is ≈1100 m and the northern edge (next to the measurement tower) is the starting point for the southward orientation of the flight legs. The locations of the meteorological tower and the Sodar are also marked in Figure 3 and are used to compared the data with the MASC-3 measurements. The “Circle” pattern is used for profiling with constant vertical ascent rate. With a large enough radius and consequently a low bank angle of the UAS, this pattern can be used for continuous profiles of wind speed, direction, temperature and other quantities. For complex terrain and inhomogeneous conditions, the ”Kite” pattern is advantageous over the standard rectangle pattern due to its lack of lateral displacement of the up- and downwind leg. However, while flying Kite patterns, the UAV spends more time in turns, and subsequently, the time spent flying measurement legs per flight is lower than with the rectangle pattern.

The Ardupilot firmware running on the Cube features automatic landings. Figure 2 shows the automatic landing process of MASC-3, which was continuously performed for nocturnal operations during the Hailuoto-II campaign. While approaching the landing spot, MASC-3 engages its flaps and reduces its altitude to 20 m above ground level (AGL). It then executes a preflare by reducing throttle and increasing the pitch angle to reduce its airspeed to 16 ms${}^{-1}$. After further descent to 8 m AGL it executes a flare with further reduction of airspeed to 12.5 ms${}^{-1}$.

The remaining altitude is reduced until touchdown with 12.5 ms${}^{-1}$ airspeed. This implemented procedure assures reliable landings of MASC-3 and therefore increases the efficiency of a measurement campaign, especially during operations at night.

### 2.3. Sensor System Setup

**Figure 4:**
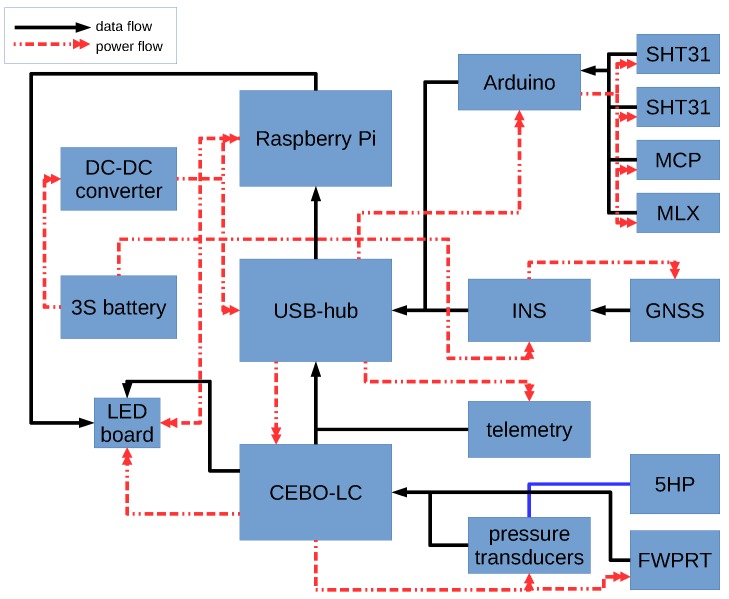
Data and power flow diagram of the MASC-3 sensor system.

Attached to the Raspberry Pi 3, the standard setup of MASC-3 has a variety of meteorological sensors and power handling devices. The flow diagram in Figure 4 shows the schematic powering and the data flow of the sensor system. The whole system is powered by a single 3S lithium polymer battery with a nominal capacity of 2700 mAh, allowing up-times of ≈4 h. The inertial navigation system (INS) Ellipse2-N from sbg-systems is directly powered by the battery. Since sensors and other periphery are running with 5V, the voltage coming from the battery is stepped done by a Traco Power (2 Ampere maximum current) DC-DC converter, providing a low noise power source. A USB Hub and the Raspberry Pi are directly powered from the 5V DC source. The USB Hub powers the CEBO-LC analogue-digital converter which handles the analogue signals and an Arduino which controls the digital sensors. The USB connections are also the data interfaces, for the CEBO-LC and the Arduino. The INS Ellipse2-N is also connected via USB to the Raspberry Pi.

The standard sensor system consists of the following sensors:

- Inertial navigation system (INS) Ellipse2-N from sbg-systems [58]; consisting of an inertial measurement unit, a GNSS receiver and an extended Kalman Filter, measuring attitude, position and velocity of MASC-3. With 3 Axis Gyroscopes, 3 Axis Accelerometers, 3 Axis Magnetometers, a pressure sensor and an external GNSS receiver, the INS has 0.1° roll and pitch accuracy, ≈0.5° heading accuracy, 0.1 ms${}^{-1}$ velocity accuracy and 2 m position accuracy. The accuracy is provided by the manufacturer and the test conditions for these specifications are proprietary and may not represent the performance during flight.
- Five-hole probe; manufactured by the Institute of Fluid Mechanics at the Technische Universität Braunschweig, Germany, measuring the flow angles and magnitude (airspeed vector) onto the probe at turbulent scales [59].
- Pressure transducers; 5× LDE-E 500, 1× LDE-E 250 for the static pressure port and a HCA0811ARG8 barometer. The differential pressure transducers are rated with an offset long term stability of ±0.05 Pa and a response time (τ63) of 5 ms.
- Fine wire platinum resistance thermometer (FWPRT); developed by Reference [60] with a 12.5 μm platinum wire, in order to measure the air temperature at turbulent scales.
- CEBO-LC from CESYS; providing an analogue-digital conversion of 14 single-ended or 7 differential analogue inputs with a measurement resolution of 16 bit. The accuracy is rated 0.005% Full Scale (typical) after Calibration and provides high-impedance operational amplifier inputs with a total sample-rate of 65 to 85 kSPS and a response-time (latency) of typically 0.9 ms and maximum 4 ms.
- SHT31 temperature and humidity sensor from Sensirion; fully calibrated, linearized, and temperature compensated digital output of temperature and relative humidity with a typical accuracy of ±2% RH and ±0.3 °C. The response time for humidity (τ63) is rated to be 8 s and the response time of the temperature (τ63) is 2 s.
- MLX90614 infrared object temperature sensor; facing downwards surface temperature measurement with a resolution of 0.02 °C and a measurement accuracy of 0.5 °C
- MCP9808 temperature sensor; additional temperature measurement for surveillance of the temperature of the electrical components of the sensor system.

The analogue signals of the turbulence measurements of the temperature and the 3D wind vector, acquired by the FWPRT and the five-hole probe together with the pressure transducers, are sampled with 500 Hz and converted by the CEBO-LC analogue-digital converter. The data stream is buffered by the CEBO-LC microcontroller, using a 32 Bit counter to ensure accurate temporal progression, and is logged by the Raspberry Pi 3. The digital sensors (SHT31, MLX, MCP) are controlled by an Arduino and logged with 10 Hz. The INS data has an update rate of 100 Hz (can be set to maximum 200 Hz) and is logged directly by the Raspberry Pi 3. Besides, a telemetry link to a laptop with a ground-station software allows the surveillance of an abstract of the data at 1 Hz.

**Figure 5:**
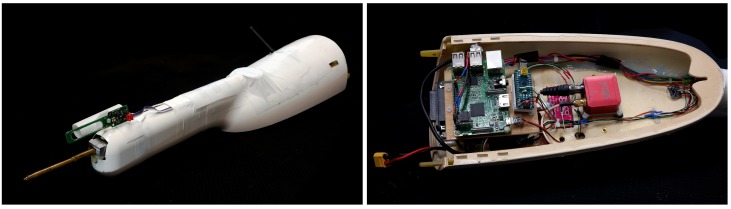
Sensor system hat (**left**) and mounted electronics inside the sensor hat (**right**).

Malfunctions of the sensors can be detected during flight and preliminary results can be plotted and, if needed, the flight strategy can be adapted. This telemetry link is provided by a small radio module (XBee) within the 2.4 Ghz band. The ground station software is also capable of calculating and displaying the potential temperature profile of the ABL on the fly, making it possible to sample more often in the layers of interest. The SHT31 sensor is mounted in two positions on the sensor system. One of them is mounted outside in a tube (see Figure 1 and Figure 5), acting as radiation shield, in order to measure the ambient air temperature and humidity alike the FWPRT. The other one is mounted inside the sensor hat to measure the temperature and humidity close to the other hardware and to monitor the temperature inside, which might be crucial in very hot or very cold conditions. The MCP9808 temperature sensor is mounted close to the pressure transducers, which are further in the front of the sensor hat, in order to monitor changes of the temperature also there. Figure 5 shows the sensor hat that is mounted on the MASC-3.

### 2.4. Sensor System Software

**Figure 6:**
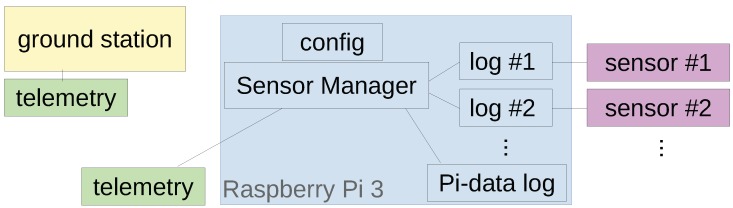
Schematic software setup of the sensor system on-board MASC-3.

The data acquisition on board the sensor hat of MASC-3 is managed by the open-source single-board computer Raspberry Pi 3. The software is designed to be a modular system that allows for switching between different sensor configurations as well as installing new sensors. A large pool of open source code examples and ready made application programming interfaces (API) allow fast implementation of new sensors. Figure 6 shows the schematic architecture of the software. The data acquisition of each individual sensor is managed by a self-contained process that connects to the sensor, logs the data on the SD card and transmits a reduced live data stream to the ground station. This design was chosen to allow for maximum freedom in choosing sensors without the restriction of being dependent on a specific programming language used by the available API of the sensor. The data acquisition software for a sensor can be written in any programming language supported by that sensors API, instead of having to create a new and potentially unreliable interface in a different programming language. The transmission of the live data stream from each sensor to the ground station software is handled by the Sensor Manager. This process is launched after the Raspberry Pi booted and starts the respective data acquisition program for each sensor in the current configuration. The live data stream from the sensor data acquisition processes is then captured and forwarded to the ground station via telemetry modules. The ground station software detects the incoming data streams and allows plotting them against each other. To ensure the modularity of the system, the logged data is not synchronized on-board. Instead, all data is oversampled and has both, the timestamp or counter of the underlying sensor, as well as the timestamp of the system time of the operating system, which itself is updated and checked against an external hardware clock. The data is synchronized during post processing by cross-checking the timestamps and counters of each of the sensors. The Raspberry Pi 3 provides data of critical parameters of the Hardware and the operating system, including for example, CPU (central processing unit) working load, CPU temperature and so forth. Along with the remaining capacity on the SD-card, which helps the ground station observer to see whether the logging process runs properly, this data is also logged and partially transmitted to the ground station.

### 2.5. Meteorological Airborne Data Analysis (MADA)

After each flight experiment, the stored data on the SD-Card of the Raspberry Pi 3 can be for example, downloaded via Ethernet. Since the CEBO-LC, the INS Ellipse2-N and the Arduino have separated log files, the data has to be merged. The first post-processing is done with the developed software MADA (Meteorological Airborne Data Analysis) which is a cumulative series of scripts based on the open source software R. The most important issue is the temporal synchronization of the data to one common time vector. The accuracy of the 32 Bit counter of the CEBO-LC and the INS, which also includes a 32 Bit counter as well as GNSS-time, ensures the accuracy of the synchronization, making the timestamps of the Raspberry Pi itself almost obsolete. Only the first and last timestamps of the Raspberry Pi inside the individual sensor log files are used to initially synchronise the logs. Subsequently the Pi time is used to double check the accurate temporal progression of the fused data. After synchronization, MADA provides scaling of the analogue sensors (e.g., FWPRT, pressure transducers, etc.) according to the calibration and data sheets. Then all data is sorted and meteorological data is calculated (e.g., air density etc.). After this pre-post-processing a first wind calculation is performed.

The 3D wind vector, using five-hole probes, is calculated by the summation of the ground speed vector of the UAS and the true airspeed vector of the UAS. The ground speed vector is directly given by the INS Ellipse2-N. By calibration, the pressure readings of the individual holes of the probe are used to estimate the true airspeed vector. To find a relationship between the measured pressure differences on the prope’s pressure holes and the airflow angles, as well as the dynamic and static pressure at any airflow angle within the calibration range, wind-tunnel calibrations were conducted. In the wind tunnel, the airspeed is set for the calibration. With dimensionless coefficients, a set of polynomial functions for the airflow angles and the dynamic and static pressure are determined. These calibration polynomials are finally used to convert the pressure readings of the measurement to the true airspeed vector of the UAS [45]. With the attitude, position and velocity of the UAS, measured by the INS, multiple coordinate transformations finally yield the wind vector. This method is widely used with UAS [18,22,47] and was originally used with manned aircraft [62]. A detailed description of the method is given in the study described in Reference [48], which also compares this direct method of the 3D wind vector measurement with simplified algorithms.

After the initial calculation of the 3D wind vector, a set of plots is printed out in order to get a first overview of the flight. Subsequently, suitable pairs of flight legs (straight, horizontal flight sections) for the wind correction [47] are identified by the software MADA. Since a misalignment between the five-hole probe’s orientation and the UAS cannot be avoided, three offset corrections for the Euler Angles ΔΦ (roll), ΔΘ (pitch) and ΔΨ (yaw or heading) must be determined. A fourth correction factor $f_{\mathrm{tas}}$ for the norm of the true airspeed vector accounts mostly for the calibration in the wind tunnel, which never matches exactly the conditions during the measurements. The assumptions for the in-flight calibration are a constant mean horizontal wind, a mean vertical wind near zero and low turbulence or turbulent transport. This allows a comparison of the wind components for two consecutive straights in opposite directions (star pattern), or identical legs in reverse direction. The correction offsets and factor for the presented flights in Section 4 were each determined with two pairs of legs in reverse direction on ≈100 m AGL. The procedure to calculate the correction factors was explained in detail in Reference [47] and analyzed with regard to the calibration of the five-hole probe and turbulence measurements in Reference [45]. If the meteorological conditions did not change substantially, the correction offsets and factor can be taken from previous flights, at least for a preliminary analysis in the field.

Afterwards, the meteorological data is processed again, including the corrections for the wind vector components. A first quality control with several plots of the measured quantities along the flight legs are printed out. Additionally, the power spectra and structure functions of the main quantities are plotted for a the quality control just after the measurements. Furthermore, vertical profiles of wind speed, wind direction and turbulence quantities are provided, containing the data of each flight leg. These quick looks are essential to get a brief overview of the meteorological conditions. An adaption of the flight patterns for consecutive flights can be considered, or a sensor malfunction can be identified. The set of plots is at hand, minutes after landing the UAV. The MADA software concept and the first analysis on sight is the foundation for a detailed post-processing of all measured data but also a key element for successful field campaigns. Uncertainty analysis of the wind vector measurement, such as the influence of the calibration procedures of the five-hole probe, airspeed variations of the UAS during the measurement, the influence of dynamic motion of the UAS and an estimation for the error propagation is given in References [45,47].

## 3. Methods and Data

An important difference, when comparing turbulence measurements with fixed-wing UAS along a straight, horizontal flight path (leg), with measurements of meteorological towers is, that the UAS transects the air with its cruising airspeed and the tower measures the air that is advected with the mean flow. Since MASC-3 is not dependant on the mean flow it is capable of gathering quasi-spatial snapshots with higher temporal resolution than the tower. The most important criteria to do so is a fast sensor, able to capture most of the energy inheriting fraction of the inertial sub-range of turbulence. The most important underlying assumption for a comparison is Taylor’s hypothesis of frozen turbulence [1], which was found to be applicable to the smallest scales of turbulence at high frequencies or low wave numbers [2]. For the larger scales, especially for atmospheric flow under the influence of the diurnal cycle, coherent structures or the variability of the geostrophic wind, differences due to production and diffusion processes of turbulence persist if a quasi-spatial snapshot is sampled with an aircraft and compared to fixed-point measuremnt with a tower [3,63]. Coming along with that, the important question of how long is long enough for a horizontal flight leg [9] when calculating turbulence statistics, causes further complexity, making comparisons between the moving UAS and a stationary tower challenging.

### 3.1. Statistical Methods

The wind vector components can be compared separately and a differentiation between the horizontal components *u* (positive eastward) and *v* (positive northward) and the vertical wind component *w* (positive when facing upwards) is insightful. The horizontal wind speed $v_{h}$ is calculated with the wind vector components *u* and *v* by

(1) $$v_{h}=\sqrt{u^{2}+v^{2}}.$$

Furthermore, the variances for the wind vector components must be compared for a validation. The variance of a variable *X* is

(2) $${\mathrm{Var}}_{X}=\sigma_{X}^{2}=\frac{1}{N-1}\sum_{i=1}^{N}{\left(X_{i}-\bar{X}\right)}^{2},$$

where *N* is the number of data points and $\bar{X}$ denotes the mean of the variable within the data window which, in this case, is the length of individual flight leg. The covariance ${\mathrm{Cov}}_{XY}$ of two variables is

(3) $${\mathrm{Cov}}_{XY}=\frac{1}{N-1}\sum_{i=1}^{N}\left(X_{i}-\bar{X}\right)\left(Y_{i}-\bar{Y}\right).$$

The turbulent kinetic energy TKE is

(4) $$\mathrm{TKE}=\frac{1}{2}\left({\mathrm{Var}}_{u}+{\mathrm{Var}}_{v}+{\mathrm{Var}}_{w}\right).$$

The integral time scale I(X) of a quantity *X* is defined by

(5) $$I\left(X\right)=\int_{0}^{\tau_{1}}\;\frac{\sigma_{X}\left(t+\tau \right)\;\sigma_{X}\left(t\right)}{\sigma_{X}^{2}}\;d\tau .$$

The integral time scale I(X) is the autocorrelation function of the variable *X* and calculated by integration from zero lag to the first crossing with zero at $\tau_{1}$ [64] and is multiplied by the mean true airspeed $\bar{|{\vec{u}}_{a}}|$, calculated for example, according to Reference [45],

(6) $$L\left(X\right)=I\left(X\right)\;|\bar{{\vec{u}}_{a}}|$$

or, respectively for the measurement tower, by the mean horizontal wind speed $\bar{v_{h}}$ in order to get the integral length scale L(X) [3,64,65,66].

The integral length scales of the horizontal wind speed $L(v_{h})$ and the vertical wind L(w) are considered in this study. The integral length scale can be interpreted as the typical size of the largest, or most energy-transporting eddy. To analyse the scale dependence of turbulence and to evaluate whether the inertial sub-range is suffiently resolved [4], spectra and structure functions of the horizontal $v_{h}$ and vertical wind *w* are analysed and compared to the measurements of the tower. The frequency spectrum—or power spectrum $S_{X}\left(f\right)$—of a quantity *X* is calculated for a time series of length Δt with the time steps *t* and after applying a Hann window by

(7) $$S_{w}\left(f\right)=\int_{0}^{\Delta t}{\mathrm{Cov}}_{XX}\left(t\right)e^{2\pi ift}\;dt=\frac{1}{\Delta t}\;\tilde{X^{*}}\left(f\right)\;\tilde{X}\left(f\right)=\frac{1}{\Delta t}{\left|\tilde{X}\left(f\right)\right|}^{2}$$

with the frequency *f*, imaginary unit *i*, covariance function from Equation (3) and the Fourier transformed frequency series $\tilde{X}\left(f\right)$ and its complex conjugate $\tilde{X^{*}}\left(f\right)$. For locally isotropic turbulence, the inertial subrange is characterized by the −5/3 slope in the spectrum. In order to compare the data of a moving UAS with the data of a stationary measurement tower, the frequency spectra $S_{X}\left(f\right)$ are transformed into wavenumber spectra $S_{X}\left(k\right)$ by

(8) $$k=\frac{2\pi f}{\bar{v}}$$

using for the transformation of the evaluated period the mean velocity $\bar{v}=\bar{v_{h}}$ of the horizontal wind speed of the measurement tower and the mean true airspeed $\bar{v}=\bar{|{\vec{u}}_{a}}|$ of the UAS measurements. The structure function $D_{X}\left(s\right)$ of a quantity *X* is calculated for a time series with *N* data points, the time steps *t* and the temporal shift or lag *s* by

(9) $$D_{X}\left(s\right)=\frac{1}{N-n}\sum_{i=1}^{N-n}{\left(X\left(t\right)-X\left(t+s\right)\right)}^{2},$$

where *n* is the number of data points associated with the lag *s*. For locally isotropic turbulence, the inertial subrange is characterized by the a 2/3 slope in the structure function.

To compare the structure function $D_{X}\left(s\right)$ of a time series between the moving UAS data and the stationary tower data, the temporal shift or lag *s* is transformed into a spacial lag *r* by

(10) $$r=s\;\bar{v}$$

also using for the transformation of the evaluated period the mean velocity $\bar{v}=\bar{v_{h}}$ measured by the tower and $\bar{v}=\bar{|{\vec{u}}_{a}}|$ measured by the UAS. The structure function and the power spectra of the horizontal wind $D_{v_{h}}$, $S_{v_{h}}$ and the vertical wind $D_{w}$, $S_{w}$ are considered in this study.

Differences concerning the fact that MASC-3 samples a quasi-spatial snapshot along a straight and horizontal flight leg with its cruising airspeed and that the stationary tower samples the advected air flow can be considered by comparing the quantities of interest for time series that have the same temporal fetch. The temporal fetch is represented by the approximated time interval for the individual measurement system during which the same volume of air was sampled. To account for that, the considered duration of the time series Δt for the comparisons in Section 4 inherit the same temporal fetch calculated by

(11) $${\Delta t}_{\mathrm{tower}}={\Delta t}_{\mathrm{UAS}}\frac{v_{\mathrm{UAS}}}{v_{\mathrm{tower}}}={\Delta t}_{\mathrm{UAS}}\frac{|\bar{{\vec{u}}_{a}}|}{\bar{v_{h}}},$$

using the mean true airspeed $|\bar{{\vec{u}}_{a}}|$ of the UAS divided by the mean horizontal wind speed $\bar{v_{h}}$, measured by the UAS.

This factorization for defining the duration of the compared time series complies with the full duration of the MASC-3 flight leg and the corresponding duration of the time series of the tower measurement ${\Delta t}_{\mathrm{tower}}$ is calculated with Equation (11).

### 3.2. Meteorological Tower and Sodar Measurements for Comparison

**Figure 7:**
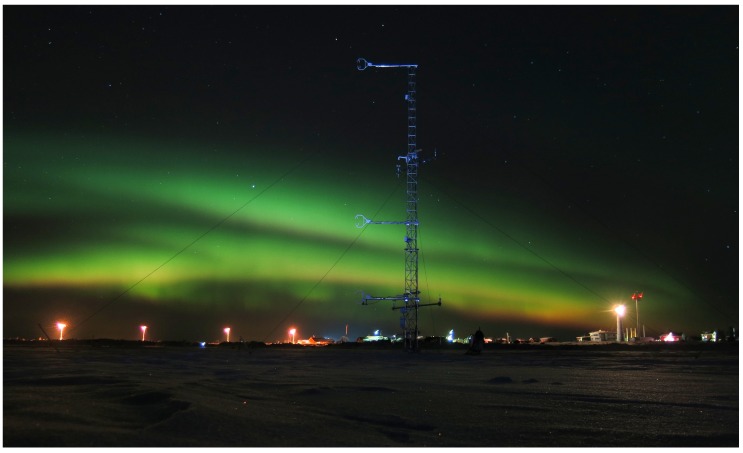
Meteorological measurement tower during the Hailuoto-II campaign. Viewing direction is north-north-east towards the harbour and the village Marjaniemi. The picture was taken by Kristine Flacké Haualand.

During the Hailuoto-II measurement campaign at the eastern coast of the north Bothnian Bay, Finland, two flight experiments were dedicated to compare the MASC-3 measurements with the meteorological tower (see Figure 7) measurements. Both flights, Flight #10 and #11 (Figure 3), were conducted on the 10 February 2018 around 17:00 (EET) and 22:00 (EET) over the completely frozen bay area west of the island Hailuoto. Civil Twilight started at 17:25 (EET) and night started at 19:21 (EET) on the measurement day.

The meteorological conditions during the evening of the 10 February 2018 were characterized by a high pressure system over Siberia and a low pressure system just south of Iceland with a relatively weak pressure gradient at our observation site. The local conditions were mostly cloudy or partially cloudy with a cloud base height below 500 m before 17:00 UTC. Between 17:00–18:30 UTC the sky opened up and became clear. Temperatures were quite moderate, slightly below freezing and decreasing throughout the evening and night. The wind direction was from south with a weak shift towards SSW and SW during the night. Relatively high wind speeds of up to 10 ms${}^{-1}$, observed at the permanent weather station at 46 m above sea level declined and stabilized at 5–7 ms${}^{-1}$ after 22:00 UTC.

The MASC-3 measurements were synchronized with the mast measurements using the Equation (11), because the aim is to sample the same volume of air with both systems. Since both flights started nearby the mast (Figure 3), the first timestamp was chosen to be the same for both systems.

**Figure 8:**
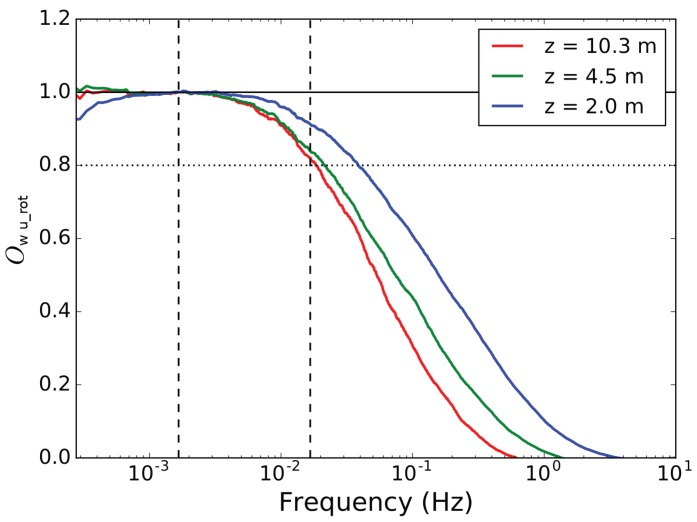
Results of the ogive test between vertical *w* and the horizontal $u_{rot}$ wind components using all observations from the three tower heights during the period 10 February 2018 14:30–22:00 UTC. Ogives are normalized by the value at the point closest to the frequency corresponding to 10 min, indicated by the first vertical dashed line from left. The second vertical dashed line represents the frequency corresponding to 60 s.

CSAT3 (Campbell Scientific, Inc., Shepshed, UK) sonic anemometers were deployed at three levels of the meteorological tower, at 2.0 m, 4.5 m and 10.3 m heights. These instruments provide measurements of the three wind velocity components and the sonic temperature at 20 Hz frequency. The data were first checked for unphysical values and spikes. The thresholds for unphysical values were ±30 ms${}^{-1}$ for horizontal wind components, ±10 ms${}^{-1}$ for the vertical wind component and ±30 °C for the sonic temperature. Spikes were detected using the method described by Reference [7]. The value of the next point in the time series was predicted based on weighted average of the last value and the mean of the last 80 values (which corresponds to a time interval of 4 s with 20 Hz sampling frequency). The weight of the last value depends on the auto-covariance between the consecutive values in the window of 80 values. If the absolute difference between the predicted and the observed value exceeds a certain threshold times the standard deviation of the last 80 values, the observation is considered as a spike. The detection algorithm was applied with a moving window of 80 values and a spike detection threshold of 4.0 and 5.5 (and an increase in threshold by 0.1 and 0.5 after each iteration, to account for the decreased standard deviation after removal of spikes) for the wind components and the sonic temperature, respectively. Spike detection was first applied forward in time and then backwards. Only those spikes that were detected as spikes from both directions were finally considered as spikes. During the selected period, 14:30–22:00 UTC on 10 February 2018, only a few (from 0 to 4 out of 540,000) individual suspicious values were detected for each variable and measurement height. These individual spikes in the 20 Hz data were replaced by linear interpolation using neighboring good quality values. After the quality control, momentum flux convergence was evaluated by ogive test [67]. Ogive function is the cumulative integral of the co-spectrum starting from the highest frequencies. The convergence is achieved when the function reaches a certain level where no more energy is gained by including larger scales. In ideal conditions, ogive function can be used to detect the location of the spectral gap between the turbulent scales and diurnal/synoptic scales. Results of the ogive method are shown in Figure 8 for the 3 different heights of the tower indicated by different colors.

The ogive functions are normalized by the value at the point closest to the frequency corresponding to 10 min period. At all levels, the ogive function reached the value 1.0, that is, the convergence, in less than 10 min. As expected, the convergence was reached faster closer to the surface than at higher levels of the tower. Further, at least 80% of the total flux was covered already within 60 s, which makes the data set suitable for comparison of turbulence measurements from the tower and MASC-3. Based on the results from the ogive test, we chose a fixed 10 min sample length for the tower measurements. For each 10-min sample, the wind components (originally in the inertial coordinate system) were rotated using 2D rotation method to align the wind components along the mean wind ($\bar{u_{rot}}$ = $\bar{v_{h}}$) and perpendicular to it ($\bar{v_{rot}}=\bar{w_{rot}}=0$). The turbulence statistics were then calculated using these rotated 10 min samples.

With the prevailing wind direction for both Flights #10/#11 of ϕ≈ 150° at the height level of the comparison between the tower and MASC-3, the flow is advected over the south-western edge of the island Hailuoto (see Figure 3). For these conditions, the shore of Hailuoto was ≈1500 m away from the tower. The shore area is not forested but the structure of the surface comprises unevenness. Generally, the structure of the surface and its roughness was not totally homogeneous. Apart from the shore of Hailuoto and the harbor, the vicinity of the measurement site was covered with isolated ice features with heights of ≤0.5 m. Close to the shore area, some bigger ice ridges of up to 3 m persisted. The footprint for both systems, MASC-3 and the tower may therefore influence the comparison.

A MFAS Sodar system (Scintec AG) was installed at the Hailuoto-II field site on the shore line (see Figure 3) with a base height of approximately 5 m above the sea level. The acoustic Sodar antenna of the MFAS consists of 64 piezo-electric transducers, emitting and receiving sound pulses at 10 different frequencies in the range 1650 Hz–2750 Hz and an output power of 7.5 W. The MFAS can emit acoustic signals in 5 different directions, vertically and tilted in N, E, S and W direction. This enables for the computation of 3-dimensional wind profiles at a vertical resolution of 10 m, ranging from 30 m to 1000 m. However the maximum range of the system was typically below 600 m. The temporal resolution of the Sodar data is 10 min, due to measurement sequence chosen for Hailuto-II. The manufacturer stated the accuracy for the wind speed and direction to be ±0.3 ms${}^{-1}$ and ±1.5° repsectively. For the comparison of the vertical profiles of the horizontal wind we chose one or two Sodar profiles, that matched the time periods of the MASC-3 ascents or descents. Only high quality Sodar data (i.e., high cumulative significance and significance density; temporal and spatial consistency) are taken into account for the analyses presented in Section 4.2.

## 4. Results

In this Section, we will first compare measurements from two horizontal low level flight legs of Flight #10 with measurements from the meteorological tower (Section 4.1). This will provide an overview of the quality of the MASC-3 data. Then, in Section 4.2, we will illustrate the potential of MASC-3 to complement meteorological mast and Sodar measurements by providing measurements from several heights of Flight #11. From these horizontal flights at multiple heights it is possible to derive profiles of mean atmospheric quantities that can be compared to the sodar measurements, as well as profiles of turbulence quantities.

### 4.1. Comparison of Measurements from MASC-3 and the Meteorological Tower

**Figure 9:**
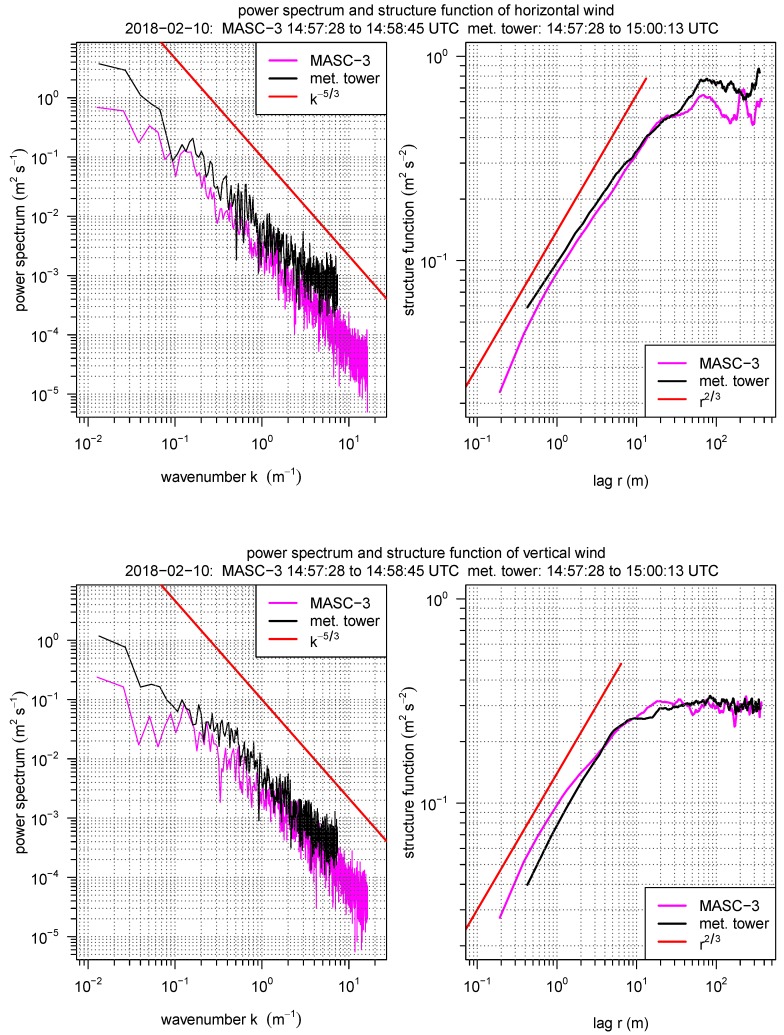
Wavenumber spectra (**left**) and structure functions (**right**) for the horizontal wind $v_{h}$ (**top**) and the vertical wind vector component *w* (**bottom**). The data of the tower at the 10.3 m level inherits a time series of ${\Delta t}_{\mathrm{tower}}=165$ s, corresponding to the fetch of the MASC-3 flight leg with a duration of ${\Delta t}_{\mathrm{UAS}}=77$ s. Flight #10 and the first leg at 11.7 m AGL is given.

**Figure 10:**
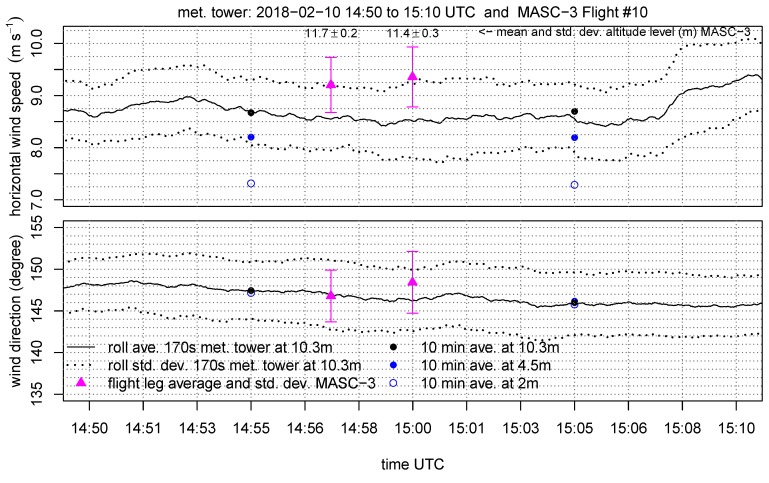
Time series of the tower with the corresponding leg averages and standard deviation of MASC-3 at the lowest flight levels for the horizontal wind $v_{h}$ (**top**) and the wind direction ϕ (**bottom**). The mean altitude and standard deviation of the individual flight leg is given for the MASC-3 data points. The data of the tower at 10.3 m is plotted as rolling (moving) average with standard deviation and a window length of ${\Delta t}_{\mathrm{tower}}=170$ s corresponding to the fetch of the MASC-3 flight legs with an average duration of ${\Delta t}_{\mathrm{UAS}}=80$ s. Furthermore the neighboring ten minute averages of the tower at all height levels are given.

**Figure 11:**
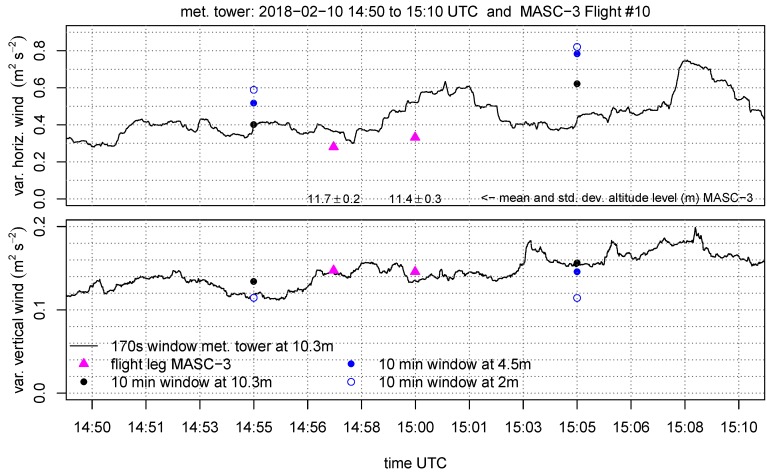
Time series of the tower with the corresponding leg averages of MASC-3 at the lowest flight levels for the variance of the horizontal wind ${\mathrm{Var}}_{v_{h}}$ (**top**) and the vertical wind component ${\mathrm{Var}}_{w}$ (**bottom**). The mean altitude and standard deviation of the individual flight leg is given for the MASC-3 data points. The data of the tower at 10.3 m is calculated on a moving window with a width of ${\Delta t}_{\mathrm{tower}}=170$ s corresponding to the fetch of the MASC-3 flight legs with an average duration of ${\Delta t}_{\mathrm{UAS}}=80$ s. Furthermore the neighboring ten minute averages of the tower at all height levels are given. The ${\mathrm{Var}}_{w}$ for the first ten minute interval of the tower at 10.3 m and 4.5 m lie on top of each other.

**Figure 12:**
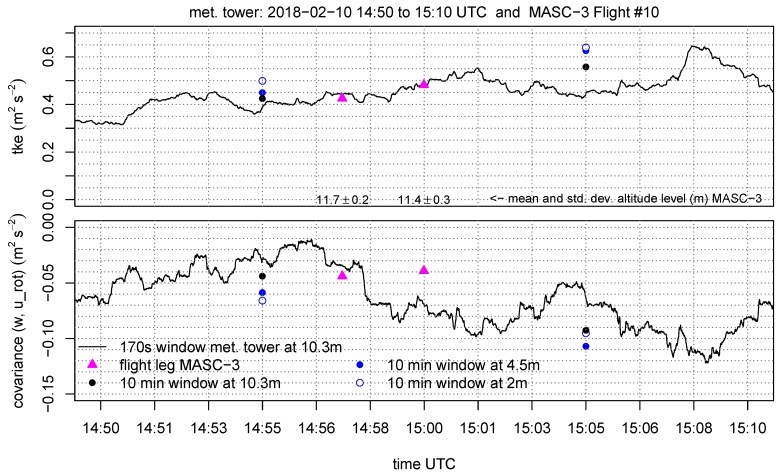
Time series of the tower with the corresponding leg averages of MASC-3 at the lowest flight levels for the turbulent kinetic energy (TKE) (**top**) and the covariance of the vertical and horizontal wind component ${\mathrm{Cov}}_{wu_{rot}}$ (**bottom**). By 2D double rotation for the tower and by coordinate transformation with the mean wind direction of the individual MASC-3 flight legs, $u_{rot}$ was aligned with the mean wind direction. The mean altitude and standard deviation of the individual flight leg is given for the MASC-3 data points. The data of the tower at 10.3 m is calculated on a moving window with the length of ${\Delta t}_{\mathrm{tower}}=170$ s corresponding to the fetch of the MASC-3 flight legs with an average duration of ${\Delta t}_{\mathrm{UAS}}=80$ s. Furthermore the neighboring ten minute averages of the tower at all height levels are given.

**Figure 13:**
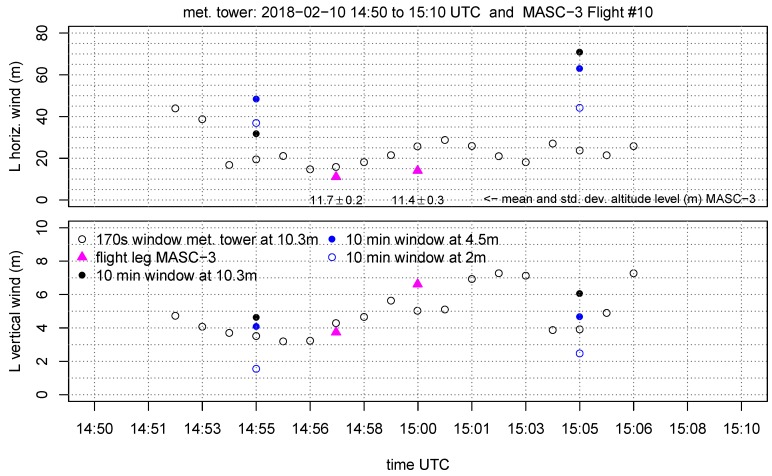
Integral length scales of the horizontal wind $L(v_{h})$ (**top**) and the the vertical wind component L(w) (**bottom**). For the tower at 10.3 m, several fractions of the time series with a duration of ${\Delta t}_{\mathrm{tower}}=170$ s, corresponding to the fetch of the MASC-3 flight legs with an average duration of ${\Delta t}_{\mathrm{UAS}}=80$ s, were used to plot the length scales alongside the values for the individual MASC-3 flight legs. The mean altitude and standard deviation of the individual flight legs are indicated. Furthermore, the integral length scales of the 10 min time series of the tower at all height levels are given.

For Flight #10, with a mean sampling time for the two flight legs of ${\Delta t}_{\mathrm{UAS}}=80$ s, the corresponding sampling time of the measurement tower is ${\Delta t}_{\mathrm{tower}}=170$ s, since the true airspeed was $|\bar{{\vec{u}}_{a}}|=19.7$ ms${}^{-1}$ and the mean horizontal wind speed $\bar{v_{h}}=9.25$ ms${}^{-1}$ (Equation (11)). The first part in Section 4.1 analyses the power spectra (see Equation (7)) and the structure function (see Equation (9)) of the horizontal and vertical wind of one flight leg of MASC-3 and the corresponding data of the measurement tower in Figure 9. To allow comparability, the frequency spectra are transformed into a wavenumber spectra, using Equation (8) and the structure functions, computed over temporal lags, are transformed into spatial legs with Equation (10). To closely compare the two measurement systems, the time series of the measurement tower is plotted and the data of the two spatially closest flight legs of Flight #10 are included in the set of plots in the Figure 10, Figure 11, Figure 12 and Figure 13. The time series inherited in the power spectra and the structure functions is the same than the first flight leg in the following set of figures.

The set of figures consist of the two neighboring sets of data points, which are the 10 min periods of the tower measurement on the three height levels (10.3 m, 4.5 m, 2 m AGL). Additionally, the data at 10.3 m AGL is plotted as moving average, variances covariance and TKE calculated on a moving window. The window size for the moving calculation of the quantities is ${\Delta t}_{\mathrm{tower}}=170$ s, allowing a close comparison with the data points of the MASC-3 measurement. Further, the integral length scales of the horizontal $L(v_{h})$ and vertical L(w) wind are plotted A moving calculation of the integral length scale according to Equation (5) is not feasible, since the autocorrelation function must be manually checked for plausibility since it may fail and not converge to a $\tau_{1}$ [64]. Therefore, a moving integral length scale is not feasible, but the actual comparison with MASC-3 and the additional calculations of *L* on time series of length ${\Delta t}_{\mathrm{tower}}=170$ s are given to analyze the temporal variability during the comparison.

The wavenumber spectra and the structure functions for the horizontal wind $v_{h}$ and the vertical wind component *w* in Figure 9 give insight in the resolution of both measurement systems. The inertial subrange of turbulence in an isotropic flow is characterized by the $k^{-5/3}$ slope in the power spectrum and by the $r^{2/3}$ slope in the structure function, indicating the ability and quality of the measurement system to resolve the spectrum of turbulent fluctuations in the atmospheric boundary layer. Generally, the discretization of the structure function is sparse towards small lags and the influence of sensor noise is better visible in the power spectrum. Vice versa, the power spectrum is sparsely discretised for small wave numbers and to study the production subrange and the onset of the inertial subrange, the structure function is beneficial.

The power spectrum of $v_{h}$ of the tower measurement is located slightly higher than the spectrum of the MASC-3 data, since the variances of the vertical wind ${\mathrm{Var}}_{v_{h}}$ (visible in Figure 11) of the tower measurement are also slightly higher. The structure functions of both time series for $v_{h}$ agree well in the inertial subrange but in the production subrange the curve of the tower data lies above the curve of MASC-3, which can also be explained by the difference in the variance measurements of both systems of ≈0.05 m${}^{2}\;$s${}^{-2}$. If the variance, as an indicator of turbulence, is higher, the spectra is located higher and the production subrange is elevated. Although only ≈2 m altitude offset persist between the sonic anemometer and the average flight level of MASC-3, this can explain the differences, since the structure of the surface layer changes strongly with height (indicated e.g., by the 10 min averages at the three tower levels in the set of plots in the Figure 10 and Figure 11). The power spectra and the production subrange in the structure functions lie close together for the vertical wind component *w*, since the variances of the vertical wind ${\mathrm{Var}}_{w}$ are almost identical. Also the gradient $\Delta {\mathrm{Var}}_{w}/\Delta z$ is smaller than $\Delta {\mathrm{Var}}_{v_{h}}/\Delta z$, when looking at the ten minute averages of the tower measurements (see Figure 11).

The ability to resolve the smallest structures can be closely compared when looking at the power spectra and towards growing wavenumbers. For $v_{h}$ and *w* and for both measurement systems, a flattening of the spectra into the horizontal, indicating sensor noise, can be observed starting from k≈ 4 m${}^{-1}$ for the tower and from k≈ 10 m${}^{-1}$ for MASC-3. Both measurement systems seem to resolve the fluctuations of *w* slightly further. The structure functions indicate, that the onset of the inertial subrange of $v_{h}$ starts at lags of ≈20 m for MASC-3 and the tower. Discrepancies can be seen in the structure functions of *w*, where the $r^{2/3}$ slope is reached only at smaller lags for MASC-3 (r≈ 20 m) than for the tower (r≈ 10 m). The inertial subrange for the vertical wind component close to the ground is shifted towards smaller structures due to the stability of the boundary layer. The length scales of the vertical wind are smaller than for the horizontal wind, which is also reflected in Figure 13. Following that, the inter-comparison of the structure functions for the MASC-3 data between $v_{h}$ and *w* does reflect this. For the tower data, this feature is less pronounced. The structure functions of $v_{h}$ and *w* of the MASC-3 data become steeper towards the lowest lags, also indicating the onset of sensor noise. The structure functions of the tower data do not indicate the onset of sensor noise as clear as the power spectra do. With sensor noise starting from k≈ 4 m${}^{-1}$ for the tower and from k≈ 10 m${}^{-1}$ for MASC-3 and by using Equation (8) it can be stated that, MASC-3 has, with 30 Hz, a significantly higher temporal resolution as the sonic anemometer with 6 Hz.

Figure 10 shows the horizontal wind $v_{h}$ and the wind direction ϕ during the period when MASC-3 performed the flight legs of Flight #10 at the lowest level. The graph consists of the 10 min averages for the tower measurement at the height levels 10.3 m, 4.5 m and 2 m AGL, as well as of the moving average and the moving standard deviation at 10.3 m of $v_{h}$ and ϕ. The wind speed, calculated from the MASC-3 flight legs, is higher than the curve of the moving average of the tower but the error bars are overlapping. One reason is the strong gradient of the horizontal wind speed, as indicated by the 10 min averages of the tower measurement. The height offset of the flight path and the highest level of the tower is only 1–2 m and the offset of $v_{h}$ is 0.75–1 ms${}^{-1}$. Although considering the gradient of the 10 min averages of the tower, a slight discrepancy of the wind speed with MASC-3 persists. The longitudinal offset of ≈100 m between the flight path and the tower and the slightly different footprint of the flow may explain this remaining small offsets. The wind direction ϕ agrees with the moving average of the tower for the first leg and differs by only 2° for the second leg. For both legs, the values of MASC-3 are within the error band of the tower measurement. Figure 10 shows, that the wind direction and speed was quite stationary during the period of comparison. The average flight level of MASC-3 with 11.7±0.2 m AGL for the first leg and 11.4±0.3 m AGL for the second leg indicate that the flight level is held precisely by the autopilot. Especially in SBL with large vertical gradients, this is important for the accuracy of the measurements with MASC-3.

The variances of the horizontal wind speed ${\mathrm{Var}}_{v_{h}}$ and the vertical wind vector component ${\mathrm{Var}}_{w}$ are given in Figure 11. The first flight leg of MASC-3 shows, with ≈0.05 m${}^{2}\;$s${}^{-2}$, a smaller value for ${\mathrm{Var}}_{v_{h}}$ than for the tower. The second flight leg has a deviation of ≈0.15 m${}^{2}\;$s${}^{-2}$. The calculation of ${\mathrm{Var}}_{v_{h}}$ on the moving window shows a temporal increase during the second flight leg, concluding that the flow field is not stationary. The deviations can be partly explained by the strong gradient, as indicated with by the 10 min values of the tower ($\Delta {\mathrm{Var}}_{v_{h}}/\Delta z$), as well as by the temporal and spatial variability. The data points of the variances of the vertical wind vector component ${\mathrm{Var}}_{w}$ agree very well with the tower measurements. This quantity is less subject to temporal change or the influence of gusts and transient motions on minute time-scales during the period of comparison, than ${\mathrm{Var}}_{v_{h}}$. Although the 10 min values of the tower also indicate, with a changing offset between the height levels (gradients $\Delta {\mathrm{Var}}_{w}/\Delta z$) between the first and the second group of values, that the state of the boundary layer changes.

The turbulent kinetic energy TKE in Figure 12 also agrees very well between the measurement systems. The temporal evolution of the structure of the boundary layer is again visible in the gradients ΔTKE/Δz of the 10 min tower measurements. The importance of applying adapted window lengths is evident, since the slight increase of TKE during the period of comparison is well represented by both systems and could not be addressed by only applying flux converged 10 min windows. This is even more important for the covariances of the vertical and horizontal wind component ${\mathrm{Cov}}_{wu_{rot}}$, since the variability is high. For both measurement systems, the coordinate systems was rotated into the mean wind direction so that the horizontal wind component $u_{h}$ is aligned with the mean wind direction. The first flight leg does agree with the tower measurement for the moving window calculation and the 10 min value. The second leg has an offset of ≈0.02 m${}^{2}\;$s${}^{-2}$. This correlates with the offset for ${\mathrm{Var}}_{v_{h}}$ in Figure 11 and can also be explained by the influence of gusts and transient motions on minute time-scales of the horizontal wind. Furthermore the spatial offset between the flight path and the measurement tower may cause these differences.

The integral length scale of the horizontal $L(v_{h})$ and vertical wind component L(w) is given in Figure 13. The integral length scale can be interpreted as the biggest scales or eddies that are inherited in the measurement [68]. For the first flight leg, both systems give $L(v_{h})\approx 15$ m but for the second flight leg, the time series of MASC-3 yields again 15 m and the tower 20 m. This offset for the second flight leg does correlate with the offsets in ${\mathrm{Var}}_{v_{h}}$ and ${\mathrm{Cov}}_{wu_{rot}}$ and leads back to previous explanation. It is remarkable, that the 10 min time series result in a negative gradient $\Delta L(v_{h})/\Delta z$ for the first 10 min period and in a positive gradient for the second 10 min period, indicating again, that a temporal evolution of the boundary layer is present. Furthermore, the 10 min time series at the 10.3 m level do not agree with the smaller window of ${\Delta t}_{\mathrm{tower}}=170$ s, indicating that the shorter time periods do not include the same spectrum of eddies. The integral length scale of the vertical wind component L(w) agrees well between the measurement systems. For the same reasons than mentioned previously, the variability is less than for $L(v_{h})$. Also the deviation between the 10 min time series and the shorter time period of ${\Delta t}_{\mathrm{tower}}=170$ s of the tower measurements is smaller (1.5–2 m). Again, the comparison during the first leg agrees better than that of the second leg, where the evolution of L(w) decreases before, during and after the comparison with the flight leg.

It is concluded that the MASC-3 measurements of mean values and statistical moments of second order agree very well with the measurements of the meteorological tower. The comparison between the stationary tower and the moving UAS is best if the temporal fetch of both systems is considered. The structure functions and spectra in Figure 9 revealed, that the spatial and temporal resolution of MASC-3 is significantly higher than for the tower. This advantage is even more important if the mean wind speed is lower. In a stably stratified boundary layer, shorter averaging periods are applicable and may even be advantageous if the fast evolution of the boundary layer is of interest. Representative calculations of statistical moments of second order were given. Although the significance of only two legs is limited, the close analysis provides a first step towards validation of the 3D wind and turbulence measurements with MASC-3.

In order to summarize the persisting error sources and uncertainties for the presented comparison with the data of MASC-3 and to provide indications for future comparisons, the following list is given.

- The remaining spatial offset between the flight path and the tower, as well as differences of the footprint cause discrepancies.
- The temporal and spatial variability of the wind field and the questionable assumption of Taylor’s hypothesis of frozen turbulence for the bigger scales of the wind field cause discrepancies.
- The measured quantities from MASC-3 do not represent the whole turbulence range and the measurements are influenced by a random error, which can be improved only by either having a larger ensemble of measurements or longer flight legs in horizontally homogeneous and stationary meteorological conditions.
- An error that is caused by the flight height persists. In sheared flow the changes in flight height and the associated changes of the turbulence regime may cause random error or bias. This depends on how the flight height changes during the flight leg and how strong the shear of the boundary layer is. If the flight height is constant on average but small variations in flight height are present, a random error must be expected. If there is a trend in flight height, or the flight height is clearly above the reference, a bias must be expected.
- Airspeed variations of MASC-3 and differences in the Reynolds number of the five hole probe’s tip between the calibration in the wind tunnel and the measurement, influence the turbulence measurements [45].
- Airspeed variations of MASC-3 during the measurement cause an uneven sampling of the turbulent structures due to the acceleration and deceleration of the UAS, cf. References [69] and [37].
- The misalignment between the five-hole probe’s orientation and MASC-3 requires three offset corrections. A forth correction factor for the norm of the true airspeed vector accounts for the differences between the airspeed of the calibration in the wind tunnel and during the measurements [45,47].
- The accuracy of the pressure and temperature sensors [47,59,60], as well as the accuracy of the INS, influence the results. The influence of the INS on the turbulence measurements with MASC-3 during dynamic motions of the UAS is especially very difficult to address and has not yet been analyzed sufficiently [45].

### 4.2. Profiles of the Atmospheric Boundary Layer with MASC-3

**Figure 14:**
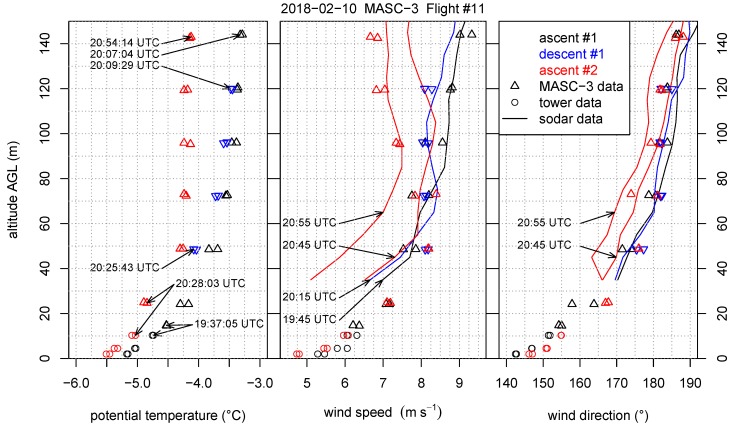
MASC-3 Flight #11 alongside the corresponding tower data and Sodar data as height profile for the potential temperature θ (**left**), the horizontal wind speed $v_{h}$ (**middle**) and the wind direction ϕ (**right**). The time series of the tower data points have a duration of ${\Delta t}_{\mathrm{tower}}=170$ s, corresponding to the fetch of the MASC-3 flight legs at the lowest levels with an average duration of ${\Delta t}_{\mathrm{UAS}}=55$ s. The timestamps of the first measurement points of each profile and the timestamps of the Sodar profiles are given.

**Figure 15:**
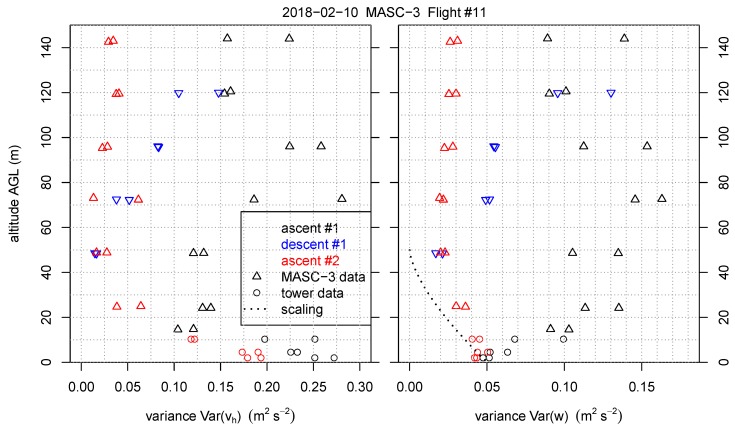
MASC-3 Flight #11 alongside the corresponding tower data as height profile for the variance of the horizontal wind speed ${\mathrm{Var}}_{v_{h}}$ (**left**) and the variance of the vertical wind speed ${\mathrm{Var}}_{w}$ (**right**). The time series of the tower data points have a duration of ${\Delta t}_{\mathrm{tower}}=170$ s, corresponding to the fetch of the MASC-3 flight legs at the lowest levels with an average duration of ${\Delta t}_{\mathrm{UAS}}=55$ s. The ${\mathrm{Var}}_{w}$ profile (**right**) inherits the scaling function.

**Figure 16:**
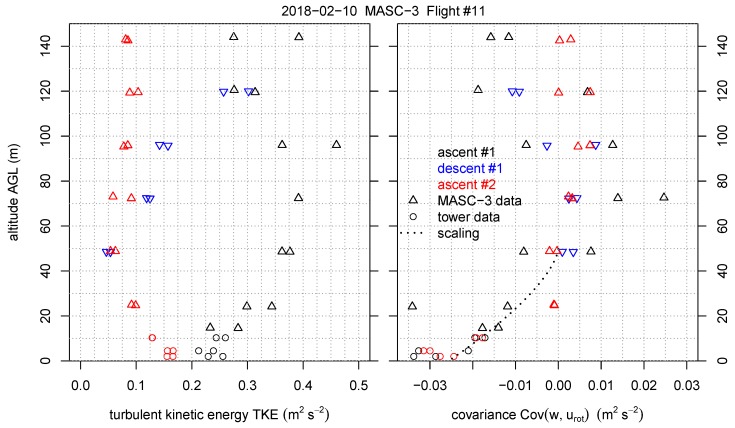
MASC-3 Flight #11 alongside the corresponding tower data as height profile for the TKE (**left**) and the covariance ${\mathrm{Cov}}_{wu_{rot}}$ (**right**) of the vertical wind *w* and the transformed vector component $u_{h}$ which is aligned with the mean wind direction. The time series of the tower data points have a duration of ${\Delta t}_{\mathrm{tower}}=170$ s, corresponding to the fetch of the MASC-3 flight legs at the lowest levels with an average duration of ${\Delta t}_{\mathrm{UAS}}=55$ s. The ${\mathrm{Cov}}_{wu_{rot}}$ profile (**right**) inherits the scaling function.

This section reverses the principle of the comparison and includes the temporally and spatially closest tower measurement periods into the height profiles of Flight #11. Figure 14 is also supplemented with the Sodar measurements and indicates the timestamps of the data of the three measurement systems. The Figure 14, Figure 15 and Figure 16 inherit the tower measurements with the equivalent timestamps of the MASC-3 legs that are closest to the tower. MASC-3 ascended, descended and ascended for a second time during Flight #11. Two racetracks were conducted at every height level, resulting in two consecutive headwind legs at each height level. During the first ascent, the lowest flight level was approximately 14 m AGL and after descending and before ascending the second time, the lowest flight level was 25 m AGL. During these lowest flight legs, the corresponding time stamps for the tower data is used for calculating and plotting the data. With $\bar{v_{h}}=6.3$ ms${}^{-1}$, $|\bar{{\vec{u}}_{a}}|=19.7$ ms${}^{-1}$ and ${\Delta t}_{\mathrm{UAS}}=55$ s for the first two legs of the first ascent, the duration of the considered time series of the tower is again ${\Delta t}_{\mathrm{tower}}=170$ s. For the second ascent, where the lowest flight level was 25 m AGL, the corresponding period of the tower was also set to ${\Delta t}_{\mathrm{tower}}=170$ s. The combined profiles of various quantities measured by the MASC-3 (triangles), the tower (circles), and Sodar (lines) are presented. Each profile took between 16 and 30 min to complete. The time difference between the first and the last triangle of a profile are summarized together with timestamp of the corresponding Sodar profile in Table 1. Each profile was flown with two racetracks, yielding two measurements per height. The first ascent started at 19:37 UTC, the descent at 20:09 UTC and the second ascent at 20:28 UTC.

Figure 14 shows the averages of potential temperature, wind speed and wind direction. Potential temperature increases with height, that is, 1.2 K in the lower 50 m, indicating the presence of a weak surface-based inversion. The first two profiles, ascent #1 and descent #1, indicate that a stable stratification persists up to 140 m; whereas the third profile, ascent #2, indicates that the atmosphere has cooled and approaches neutral stratification above 50 m. Furthermore, in the lower 50 m all flight patterns show a decrease in wind speed with height of about 3 ms${}^{-1}$ together with a change in wind direction of about 40°. The first two MASC profiles agree well with the corresponding Sodar profiles of wind speed and direction at 19:45 UTC and 20:15 UTC, whereas ascent #2 reveals for the wind speed features of both the Sodar profiles taken at 20:45 UTC and 20:55 UTC. The MASC-3 data at levels below 80 m are closer to the Sodar profile from 20:45 UTC. Above this level data are in good agreement with the Sodar data profile from 20:55 UTC. This case indicates that what at first sight appears to be a jet like feature, observed during ascent #2, is in fact the result of a strong instationarity related to a decrease in wind speed during the time it took to complete the profile. The change in wind speed and wind direction occurred relatively sudden, which explains why the red triangles at 70 m are further apart from each other than at the other levels. The wind direction profiles of the Sodar measurements during ascent #2 deviate slightly more from the MASC-3 profiles than for ascent #1 and descent #1. Further, the turning of the wind direction measured by the tower and the lowest flight legs of MASC-3 between ascent #1 and ascent #2 was oppositely measured by the Sodar profiles above.

During the whole flight, a stable boundary layer was present, but surface observations reveal that turbulence conditions were not stationary. Around 18:30 UTC clouds enter the area. Long-wave incoming radiation increases from 220 W m${}^{-2}$ to 280 W m${}^{-2}$ around 19:00 UTC, and recovers to its original values just after 20:00 UTC. At the same time, the sensible heat flux at 2 m height increases from −25 W m${}^{-2}$ at 18:00 UTC to 0 W m${}^{-2}$ at 19:00 UTC, and decreases to −20 W m${}^{-2}$ at 20:00 UTC. At 10 m height, the sensible heat flux also increased to 0 W m${}^{-2}$ at 18:00 UTC; but during the cloud free periods, the magnitude of the flux in 10 m height was about 5 W m${}^{-2}$ smaller than at 2 m height. Furthermore, stability at 2 m height was constant around 0.05 and, whereas at 10 m height values decreased from 0.4 at 18:00 UTC to 0.05 at 19:00 UTC, recovering to 0.4 at 20:00 UTC. Finally, the friction velocity, $u_{*}$, steadily decreased from 0.25 ms${}^{-1}$ to 0.16 ms${}^{-1}$ during this time period.

To get more insight in the atmospheric structure for this specific situation, the MASC-3 measurements allow to consider second-order moments as well. Figure 15 and Figure 16 present variances of horizontal (${\mathrm{Var}}_{v_{h}}$) and vertical wind speed (${\mathrm{Var}}_{w}$), as well as turbulent kinetic energy (TKE) and the covariance of horizontal and vertical winds ${\mathrm{Cov}}_{wu_{rot}}$. Note that also these variables represent time averages for the tower data of ${\Delta t}_{\mathrm{tower}}=170$ s. This is a rather short averaging interval for second-order moments but it contains 90% of the relevant information as shown by the Ogives in Figure 8 [70,71]. Furthermore, profile scaling functions of ${\mathrm{Var}}_{w}$ and ${\mathrm{Cov}}_{wu_{rot}}$ by Reference [72] are plotted. The scaling function for ${\mathrm{Cov}}_{wu_{rot}}$ is given by

(12) $${\mathrm{Cov}}_{wu_{rot}}=u_{*}^{2}\;{\left(1-\frac{z}{h}\right)}^{\frac{3}{2}}$$

and the scaling of the variance of the vertical wind vector component ${\mathrm{Var}}_{w}$ is given by

(13) $${\mathrm{Var}}_{w}=1.96\;u_{*}^{2}\;{\left(1-\frac{z}{h}\right)}^{\frac{3}{2}}$$

where *h* is the estimated height of the boundary layer. For this situation, we used $u_{*}=0.16$ ms${}^{-1}$ based on the tower data at 2 m and h=50 m. The main assumptions for this model are a stationary boundary layer with constant Richardson and Richardson flux numbers [72].

Figure 15 shows that ${\mathrm{Var}}_{v_{h}}$ is 0.20–0.25 m${}^{2}\;$s${}^{-2}$ at the surface and decreases with height in the lower 60 m AGL. Above this level, the first two profiles show increasing ${\mathrm{Var}}_{v_{h}}$, whereas the third profile remains constant with height. Also for ${\mathrm{Var}}_{w}$ an apparent difference between the first and the last profile exist. The first profile shows an increase in height in the lower 60 m AGL, whereas the third profile shows a decrease ${\mathrm{Var}}_{w}$ following the scaling function from Reference [72] presented in Equation (13). The profiles of TKE and ${\mathrm{Cov}}_{wu_{rot}}$ as shown in Figure 16 are consistent with this picture. In the last profile TKE decreases in the lower 60 m AGL, whereas in the first profile TKE increases. The ${\mathrm{Cov}}_{wu_{rot}}$, which is aligned in the mean wind and thus represents $u_{*}$, seems to follow the scaling profile of Nieuwstadt given in Equation (12) in all cases. Nevertheless, the first profile shows a greater spread between the two flight legs and suggests a maximum of ${\mathrm{Cov}}_{wu_{rot}}$ at about 70 m AGL.

These data show that during the cloudy atmospheric conditions the boundary layer is not in balance with the surface, that is, conditions are non-stationary. Ascent #1 took place in the period when the clouds were leaving the area. The radiative cooling starts to enhance the magnitude from the surface fluxes, but at greater heights turbulence is still more active due to the previously existent neutral conditions. One may argue that this profile suggests the existance of a so-called upside-down boundary layer, that is, a boundary-layer containing an elevated shear layer. The profile of ${\mathrm{Var}}_{w}$ shows a maximum at about 70 m [73] and TKE increases with height [52]. However, since conditions are non-stationary, we rather relate this elevated shear layer to the onset of radiative cooling at the surface than to an upside down boundary layer with an elevated source of turbulence cf. Reference [52,73].

Further scaling methods were not found to be applicable.

After an hour without clouds, the situation has become more stationary and profiles suggest that turbulence is now mainly confined to the surface. The potential temperature approaches the neutral stratification at heights above 60 m [68]. Note that since the wind suddenly reduced during the measurements, there is no real jet, which explains why ${\mathrm{Cov}}_{wu_{rot}}$ is not >0 above 60 m for ascent #2 cf. Reference [74]. Furthermore, turbulence parameters follow the scaling laws from Reference [72] and TKE and ${\mathrm{Var}}_{w}$ are largest close to the surface [52,73]. As such, the boundary layer classification, may be considered a weakly stable boundary layer in the transition regime, that is, no constant flux layer and stability >0.1 [75].

We conclude that the MASC-3 measurements agree well with measurements of the meteorological tower and the Sodar and the combination of these measurement systems captures the interactive nature of the stable boundary layer well. The relatively long sampling time for a full ABL profile, consisting of several straight and vertically stacked legs, may however cause misleading interpretations when sampled under conditions with strong instationarity. For such cases, additional boundary layer remote sensing systems such as Sodar are highly valuable.

## 5. Conclusions

The recent mark of the Multi-Purpose Airborne Sensor Carrier MASC-3 improved the turbulent 3D wind vector measurement and gained endurance, since the flight mechanical performance of the wing design with a high lift/drag ratio and the streamlined design is less susceptible to turbulence. The influence on the location of the sensors was minimized by locating the engine behind the tail unit. The fuselage and the installed sensor hat, as well as the modular software architecture of the data acquisition system, allow for different payloads and a variety of applications that can be supplemented to the turbulent 3D wind vector measurement. The autopilot system and the durable airframe can be deployed in polar conditions and provides manifold maneuverability including a multitude of flight patterns for different missions, as well as automatic landing. The precision and repeatability of the Pixhawk 2.1 autopilot ensures the quality of turbulence measurements in the atmospheric boundary layer. The telemetry of the autopilot and the sensor system, as well as the post-processing software MADA, provide insight of the prevailing conditions on sight and enable interactive and adjusted measurement campaigns. Two flight experiments in a SBL and a close comparison with a meteorological measurement tower, equipped with sonic anemometers, depicted the capabilities of MASC-3. Beside mean values, MASC-3 measurements allow second-order statistical moments, even suitable for estimating the turbulence regimes of SBL, where small differences distinguish between important characteristics of the SBL. The close comparison with the data of the measurement tower showed, that variances, covariances, turbulent kinetic energy and the integral length scale can be reliably estimated and agree well. With MASC-3 and its sensor system, the turbulent structure of the ABL can be sampled faster and with higher resolution than standard sonic anemometers mounted on a measurement tower. Considering the individual fetch of a stationary measurement system and a moving UAS, the systems can be plotted together with continuous profiling systems, such as Sodar to depict fast evolving SBL. Due to the ability to transect the ABL, shorter averaging intervals for second-order moments are applicable when compared to stationary measurement systems, especially if the mean flow is weak and the advection over the stationary sensors is small. The temporal evolution and transition phases between turbulence regimes can be captured with higher resolution and thus, MASC-3 is a valuable addition to meteorological towers and Sodar measurements when investigating the interactive nature of the stable boundary layer.

## Figures and Tables

**Table 1:** Timestamps (UTC) and duration of the profiles of MASC-3 and the corresponding timestamp of the Sodar measurement for Flight #11.

Flight #11	Start [hh:mm:ss]	End [hh:mm:ss]	Duration [mm:ss]	Sodar Profile [hh:mm]
ascent #1	19:37:05	20:07:04	29:59	19:45
descent #1	20:09:29	20:25:43	16:14	20:15
ascent #2	20:28:03	20:54:14	26:11	20:45 and 20:55
